# There’s life in the old dog yet: immunochemotherapy in Waldenström’s macroglobulinemia

**DOI:** 10.1038/s41375-024-02195-w

**Published:** 2024-03-07

**Authors:** Efstathios Kastritis, Christian Buske

**Affiliations:** 1 Department of Clinical Therapeutics, National and Kapodistrian University of Athens, School of Medicine, Athens, Greece; 2 Institute of Experimental Cancer Research, University Hospital Ulm, Ulm, Germany; 3 Department of Internal Medicine III, University Hospital Ulm, Ulm, Germany

**Keywords:** Lymphoma, Haematological cancer

Waldenström’s macroglobulinemia (WM) is an uncommon lymphoma with unique features, characterized by the accumulation of lymphoplasmacytic cells in the bone marrow that produce monoclonal IgM [1]. There has been considerable progress in the clinical management of this disease fueled by the introduction of covalent BTK inhibitors (BTKis) such as ibrutinib or zanubrutinib in the recent years. Based on their efficacy ibrutinib with or without rituximab and zanubrutinib as single agent are approved for the treatment of WM in many countries worldwide. Since then, the treatment landscape has substantially changed with rituximab/chemotherapy and BTKi being the two major treatment concepts prevailing for first line treatment of WM, both concepts being recommended side by side in national and international guidelines [2, 3]. As in other B-cell lymphomas there is the vision in WM to move toward a chemotherapy-free treatment landscape, similar to what has been achieved in chronic lymphocytic leukemia, for which chemotherapy is only used as exception nowadays [4].

In this issue de Tute et al. nicely demonstrate that optimized immunochemotherapy is a highly effective and well tolerated treatment in WM and that we are still far away from the point where we could afford to neglect this treatment concept and to rely solely on chemotherapy-free treatments. In this phase II study treatment naïve patients with WM were treated with bortezomib, cyclophosphamide and rituximab (BRC) for a maximum of 6 cycles. Bortezomib was given subcutaneously on a weekly basis to avoid neurotoxicity and rituximab application was limited to cycle 2 and 5 with four weekly dosing. This mild immunochemotherapy induced an overall response rate of 97.6% and at least a PR in 81.4% of patients. Most importantly, 5-year PFS was 65.5% and 5-year time-to-next-treatment (TTNT) rate was 79.4% with a 5-year OS of 95.0%. These are excellent results, considering the age and risk characteristics of the patients in the study, and are in line with a recently published large randomized phase II study of the European Consortium for Waldenström’s macroglobulinemia (ECWM) comparing dexamethasone, rituximab and cyclophosphamide (DRC) with Bortezomib-DRC in the frontline setting [5]. In addition, the toxicity of BRC was low and manageable, including relatively low neuropathy rates, probably due to the optimization of bortezomib administration. Thus, it is important to note that rituximab/chemotherapies are highly active, fixed duration regimens with a well acceptable cost-effectiveness ratio, avoiding continuous treatment and the associated cumulative toxicity. Trials prospectively comparing BTKis with immunochemotherapy head-to-head are lacking and historical comparisons have to be handled with caution. BTKis, either first (ibrutinib) or second generation (acalabrutinib and zanubrutinib) BTKis, are the most active single agents in WM. These orally administrated drugs are rapidly acting, and have a toxicity profile that are favorable in most patients. However, they require continuous therapy, their interruption may be associated with rebound phenomena and although response rates are high, complete responses are uncommon. In addition, their efficacy is reduced in non-*MYD88*^*L265P*^ and among patients carrying *CXCR4*^*WHIM*^, while their toxicity in elderly patients is non-trivial, with arrhythmias, immunosuppression, hypertension and bleeding diathesis, along with drug interactions being of concern, especially for 1st generation BTKis [2, 6]. In treatment-naïve WM, single agent ibrutinib was associated with a 4-year PFS rate of 76%, which was genotype dependent (59% for those with vs. 92% for those without CXCR4 mutations). Similar PFS rates for the total patient group were also seen in patients that received ibrutinib with rituximab in the iNNOVATE study or ibrutinib or zanubrutinib monotherapy in the ASPEN trial [7, 8]. Taking these results in mind, we have to admit that the results achieved with BRC and presented by de Tute et al. in this issue are excellent and offer the vast majority of patients a treatment free period until salvage therapy in contrast to continuous treatments, which lack this possibility conceptually. A major step forward will be to develop fixed duration chemotherapy-free approaches and to compare them to rituximab/chemotherapy in randomized trials. Such a study will be initiated soon by the ECWM, randomizing 6 cycles of DRC vs. fixed duration venetoclax/rituximab in treatment naïve WM (NCT05099471).

The second important aspect of the study presented by de Tute et al. in this issue is the establishment of an important early parameter which closely correlate with treatment outcome: using flow cytometry with a sensitivity of 0.004% the authors measured WM specific B-cell depletion in the BM after 3 cycles and at the end of treatment and found a high correlation between WM B-cell depletion and PFS, remaining independent after adjusting for baseline stratification factors or response, the latter one taking traditionally reduction of IgM serum levels as key parameter into account. Thus, at the end of BRC therapy, 50% of evaluated patients had no detectable B-cells, although several of them (12/38, 32%) were in either partial or minor remission, so they still had significant amounts of circulating IgM, with only 1 patient in CR and 6/38 in VGPR. Undetectable B-cells in the BM after therapy was associated with an excellent PFS at 3 years of 94.7% vs. 63.2%, respectively, with a HR of 0.06 (% CI: 0.01–0.47, *p* ≤ 0.001). As the flow cytometry used in this study did not comprise the malignant plasma cell compartment, these results underline the relevance of the WM B-cell compartment for disease progression. It furthermore highlights, that elimination or at least substantial reduction of WM B-cells should be a major treatment goal in this disease. Minimal residual disease (MRD) could be also a guide for more risk adapted treatments with BTKis, helping to identify patients with low risk of disease relapse after stopping the inhibitor. Studies testing this prospectively are not available yet, but MRD evaluation should be part of future clinical trials to understand better to which extent quantification of MRD is able to personalize treatment in WM. There are different molecular techniques present to measure MRD, but flow cytometry has the charm to be accessible for many centers on site with the potential to serve as a widely applicable tool for MRD-guided treatment in WM [9–11].
